# Correction: First description of a herpesvirus infection in genus Lepus

**DOI:** 10.1371/journal.pone.0233799

**Published:** 2020-05-21

**Authors:** The PLOS ONE Staff

The first author's initials appear incorrectly in the citation. The correct citation is: Abade dos Santos FA, Monteiro M, Pinto A, Carvalho CL, Peleteiro MC, Carvalho P, et al. (2020) First description of a herpesvirus infection in genus Lepus. PLoS ONE 15(4): e0231795. https://doi.org/10.1371/journal.pone.0231795.

There are errors in the Author Contributions. The correct contributions are:

**Conceptualization:** F. A. Abade dos Santos, M. D. Duarte

**Data curation:** F. A. Abade dos Santos, M. Monteiro, C. L. Carvalho, M. D. Duarte

**Formal analysis:** F. A. Abade dos Santos, A. Pinto, C. L. Carvalho

**Funding acquisition:** M. C. Peleteiro, M. D. Duarte

**Investigation:** F. A. Abade dos Santos, M. Monteiro, A. Pinto, C. L. Carvalho, M. D. Duarte

**Methodology:** F. A. Abade dos Santos, C. L. Carvalho, M. D. Duarte

**Project administration:** F. A. Abade dos Santos, M. C. Peleteiro, M. D. Duarte

**Resources:** F. A. Abade dos Santos, M. Monteiro, A. Pinto, P. Carvalho, P. Mendonça, T. Carvalho, M. D. Duarte

**Supervision:** M. C. Peleteiro, M. D. Duarte

**Validation:** F. A. Abade dos Santos

**Visualization:** F. A. Abade dos Santos, M. Monteiro, A. Pinto, P. Carvalho, P. Mendonça, T. Carvalho

**Writing–original draft:** F. A. Abade dos Santos, M. D. Duarte

**Writing–review & editing:** F. A. Abade dos Santos, C. L. Carvalho, M. C. Peleteiro, M. D. Duarte

No authors contributed to Software.

Fig 8 is incorrect. The publisher apologizes for the error. The authors have provided a corrected version here.

**Fig 8:**
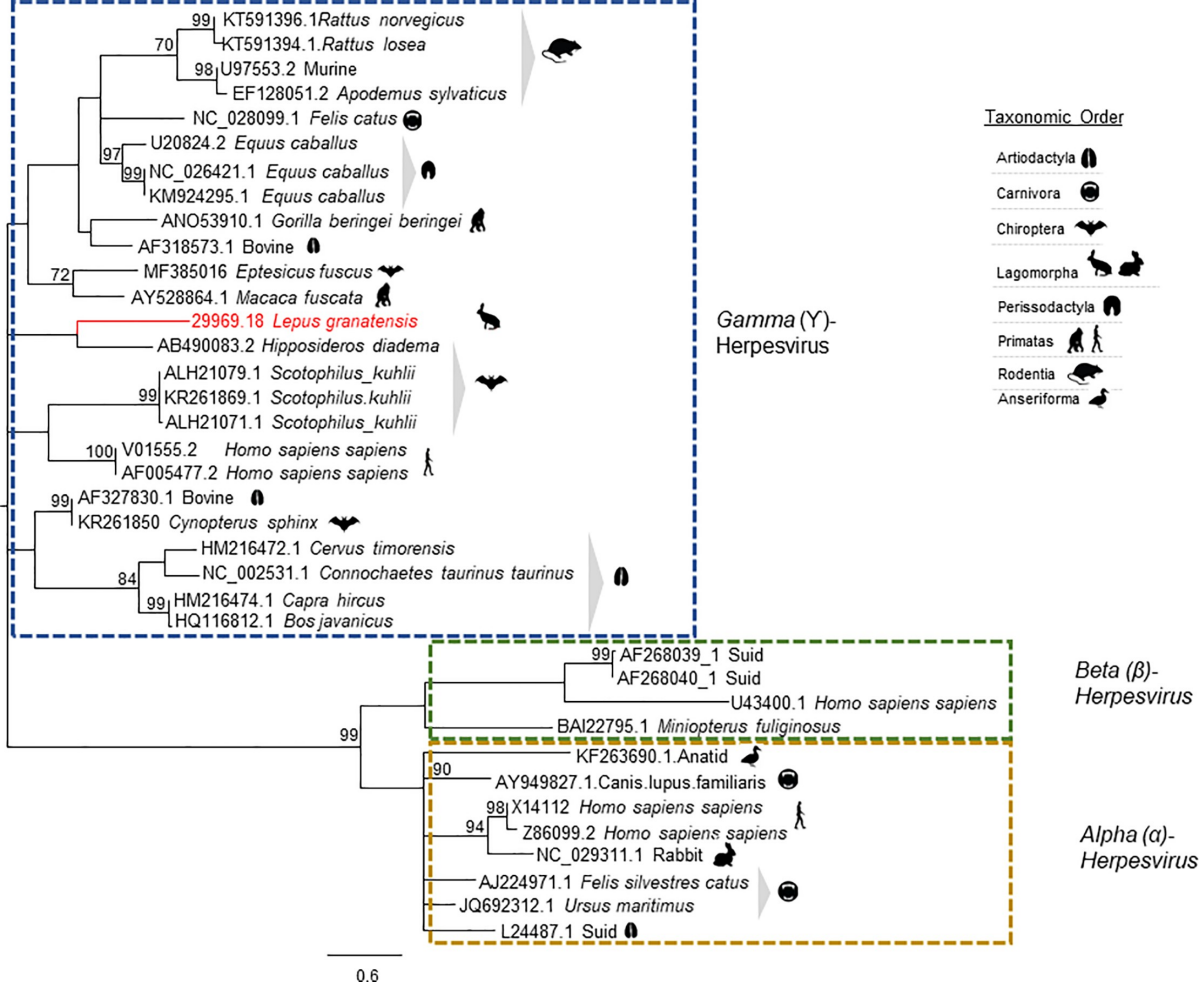
Phylogenetic analysis based on 37 partial *DNA Polymerase* amino acid sequences of herpesviruses from several vertebrate species. The access number of the nucleotide sequences from which the amino acid sequences were deduced are given. The tree with the highest log likelihood (-2432.55) is shown. The LG+G+I model considering 5 categories, [+G] parameter of 0,9929 and [+I] of 11,47% sites was used. The tree is drawn to scale, with branch lengths measured in the number of substitutions per site. There were a total of 60 positions in the final dataset. Robustness of the tree nodes was assessed by bootstrapping 1000 times. Only bootstrap values ≥70 are shown. The evolutionary analyses were conducted in MEGA X [18] and the phylogenetic tree was edited in the Figtree software version 1.4.0.
