# Supplementary material for: First description of a herpesvirus infection in genus Lepus
Source: PLoS One. 2020 Apr 17;15(4):e0231795. doi: 10.1371/journal.pone.0231795 (PMC7164596; doi:10.1371/journal.pone.0231795)
Supplement: Attachment — Submitted filename: Responses _to_the_Reviewers.pdf [file pone.0231795.s003.pdf]

Response to Reviewers

1. Please ensure that your manuscript meets PLOS ONE's style requirements, including those for file naming. The PLOS ONE style templates can be found at http://www.plosone.org/attachments/PLOSOne_formatting_sample_main_body.pdf
and
http://www.plosone.org/attachments/PLOSOne_formatting_sample_title_authors_affiliations.pdf

2. In your Methods section, please give the sources of any cell lines used in your study.
Done.

3. In your Methods section, please provide additional location information, including geographic coordinates for the sampling locations/data set if available.
Done. A new figure (Figure 1) was added to the text showing the geographic origin of the samples.

4. In your Methods section, please provide additional information regarding the permits you obtained for the work. Please ensure you have included the full name of the authority that approved the field site access and, if no permits were required, a brief statement explaining why.
Reference to the scope of the sampling and supporting legislation was included.

5. Please include a separate caption for each figure in your manuscript.
Done.

6. Please include a copy of Table 2 which you refer to in your text on page 8-9.
Done.

<u>COMMENTS TO THE AUTHOR</u>

Reviewer #1: Review comments for the manuscript number: PONE-D-20-00203 In the present manuscript, authors have identified a new herpesvirus Leporid gammaherpesvirus 5 (LeHV-5), in genus Lepus. They confirmed the presence of herpesvirus in 13 MYXV-positive hares by PCR and sequencing analysis.
However, I would like to suggest authors to pay attention to following

1. Table 1 has not been numbered correctly.
The content and format of Table 1 was revised. LeHV-5 also included to allow comparing all the leporid herpesvirus described so far.

2. Table 2 is missing.
Table 2 was included.

3. Figure legend must be little more descriptive 4.
Legend was re-written.

Figures 6, 7, 8 are missing 5.
All figures were included in this reviewed version of the manuscript.

Bacteriological and parasitological examination method should have had been described in detail under methodology section.
More detailed information was added to the manuscript as requested.

6. Description of figure 2 under the heading Electron microscopy is confusing.
Reference to Figure 2 was moved within the sentence.

7. Results for epithelial and stroma cell of the eyelid are not shown but discussed. Either show the results or don't discuss.
At this time, the figure has not enough quality to be include. If Reviewer #1 agrees, we would like to remove this sentence from the Results section and mentioned this finding in the Discussion.

8. There are few spelling mistakes .
We thank Reviewer #2 for correcting these mistakes.

Reviewer #2: Dos Santos et al describe the detection of a yet unknown gammaherpesvirus in the Iberian hare by pathology, electron microscopy, PCR, sequencing, and phylogenetic analysis. The manuscript is of interest for virologists and readers interested in hares and their diseases. The manuscript is well structured, but contains a number of mistakes, misleading phrases which should be eliminated (see accompanying word document).
Thank you for the time revising the language and content of our manuscript.

The main criticism pertains the phylogenetic analysis. Many branches of the trees are not statistically well supported. This is not surprising as the authors could only use the sequence that was derived from the generic PCR, i.e., appr. 175 bp, without the primer binding sequences (these have to be removed). It is important to perform phylogenetic analysis with extended sequences that result in statistically well supported trees. Methods to extend gammaherpesvirus sequences from the region of generic DPOL PCR into the coding sequence of glycoprotein B were published in a number of papers (e.g. Journal of Virology, 82(7), 3509-3516.). In the revised manuscript the authors should include statistically well supported trees.
Thank you for point out this mistake. When preparing the alignments, the primer sequences were quite useful for the splicing, and we forgot to remove then.
As indicated, the 5’ and 3’ ends of the sequences corresponding to the primers, were removed and the trees reconstructed. As expected no significant phylogeny changes were observed.

We agree with Reviewer#2, that the region is quite small and that extending the plylogenetic analysis to a wider region would produce more robust data. However, most of the sequences available in the GenBank are also very short, and refer exactly to this region. In fact, given the high variability found in herpesviruses, the nested PCR developed by Van Devanter et al, is commonly used by many researchers, limiting the sequences available to the size of the second PCR-amplicon (≤ 220 nt long). That is the case of the sequences KR261864 and KR261869, both from *Scotophilus kuhlii*, the herpesviruses more closely related with LeHV-5, but also KT591396 from *Rattus norvegicus*, and U97553 from Murine.
Consequently, for the purpose of our phylogenetic analysis, extending the sequence would also reduce tremendously the number of herpesvirus hosts included.
Also, as referred in the manuscript (Van Devanter et al, 1996), this small region has discriminatory power for phylogenetic inferences.

Responses to Reviewer #3

1. *** Sentence in yellow is misleading*** (Over the years, the Iberian hare has been unaffected by viral diseases that, alongside environmental and anthropogenic factors, led to the drastic decline of the wild rabbit).

Sentence was rephrased to "Contrarily to the wild rabbit, which drastic decline has been linked, among other factors, to viral epizooties, until recently, the Iberian hare was not affected by viral diseases. Environmental and anthropogenic factors, however, have had a negative impact on both hare and wild-rabbit populations."

2. (revised on ***???*** Jin et al.,2008) Of these, the most common naturally occurring herpesvirus infections identified in rabbits are LHV-2 and LHV-3, which alongside LHV-1 belong to the Gammaherpesvirinae subfamily.

We rewrote to "Of these, the most common naturally occurring herpesvirus infections identified in rabbits are LeHV-2 and LeHV-3 (reviewed by [9]), which alongside LeHV-1 belong to the Gammaherpesvirinae subfamily."

3. ***MHV68 is used as a model virus since many years***(The lack of a suitable animal model …)

Thank you for pointing out this mistake. The sentence was erased.

4. ***spell out*** (For histopathology, skin and genitalia fragments were fixated in 10% neutral buffered formalin, routinely paraffin embedded, sectioned at 4 µm, and stained with <u>H&E</u>)

Done

5. ***what is this?***(From these <u>hunted specimens</u>, no genitalia/skin samples were available for histopathology. Six hares showed doubtful results).

Sentence was changed to "Herpesvirus-DNA was also detected by PCR in the liver, spleen and lung samples of 41.2% (7/17) of the apparently healthy hunted hares that tested negative for MYXV. From this group of hares, no genitalia/skin samples were available for histopathology."

6. ***The PCR products are of appr. 225 bp length. But after subtraction of the primer sequences, the novel sequence has a length of appr 175 bp only !*** (To refine the phylogenetic analysis with regards to gammahespesviruses (Figure 8), we explored the nucleotide variability among this group, using a second set of 25 herpesviruses sequences from orders Artiodactyla, Carnivora, Chiroptera, Lagomorpha, Perissodactyla, Primates and Rodentia. The accession numbers of the original sequences from which the DNA Polymerase 225 nt long sequences were edited, are indicated in Figure 8)

Thank you for point out this mistake. When preparing the alignments, the primer sequences were quite useful for the splicing, and we forgot to remove then.

As indicated, the 5' and 3' ends of the sequences corresponding to the primers, were removed and the trees reconstructed. As expected no significant phylogeny changes were observed.

7. ***None oft he LHVs is currrently classified as species by ICTV***(According with the most recent International Committee on Taxonomy of Viruses (ICTV) guidelines for classification of viruses, we propose to name this virus species leporid gammaherpesvirus 5 (LeHV-5), following the rabbit alphaherpesvirus 4 (LHV-4), although we cannot suggest a genus for LeHV-5).
We rephrased the sentence. According to the 2018b Guidelines of ICTV, LHV-4 was included as a specie of an unclassified genus. Otherwise, LHV-1, LHV-2 and LHV-3 were not yet classified.

8. ***??? Cercopithecine herpesvirus-1 is in the Alphaherpesvirinae! There is no zoonotic murine gammaherpesvirus***, (Despite LeHV-5 not appearing to multiply in human (Hela) and primate (Vero) cells, because gammaherpesviruses may be zoonotic, as is the case of cercopithecine herpesvirus-1 and murine gamma herpesvirus13 isolation attempts was performed under BSL-2 conditions.)
Thank you for pointing out this mistake. According with what is available in the literature, we rephrased the sentence to " Despite LeHV-5 seems unable to multiply in human (Hela) and primate (Vero) cells, given the zoonotic potential of some animal herpesviruses, as the case of the cercopithecine alphaherpesvirus 1 [20] and the murine gamma herpesvirus 68 [21], all isolation attempts were carried out in BSL-2 conditions."

9. *** Please rephrase *** (This study describes the detection of a new gammaherpesvirus in the genus Lepus that, according tophylogenetic analysis, is most similar to bat and rodent gammaherpesviruses.)
Done
10. ***for what?***(However, this value may be an underestimation given that the tropism of LeHV-5 is still unknown, and consequently the tissue samples used for diagnosis may have been inadequate)
The sentence was rewritten
