# Supplementary material for: First description of a herpesvirus infection in genus Lepus
Source: PLoS One. 2020 Apr 17;15(4):e0231795. doi: 10.1371/journal.pone.0231795 (PMC7164596; doi:10.1371/journal.pone.0231795)
Supplement: Attachment — Submitted filename: Response_Editor.pdf [file pone.0231795.s004.pdf]

Dear Editor
Dr. Binod Kumar

We were pleased to see that Reviewer#1 is satisfied with the corrections done in the second version of our manuscript.
We would like to emphasise that at the main outcome of our manuscript is the demonstration that a potentially pathogenic *gammaherpesvirus* is circulating the Iberian hare, which we believe we have fully accomplished through microscopic, molecular and histopathological evidences.
Regarding the characterisation of the new LeHV-5, besides sequencing and comparison to the HV presently known by BLAST analysis, we believe that presenting a phylogenetic analysis, even if preliminary, is important and relevant.
Therefore, to respond to Reviewer #2 concerns, we reformulated the phylogenetic analysis by obtaining the nucleotide sequence of the gB gene from 3 LeHV-5 strains. This 453 bp sequence was used to construct an aa phylogenetic tree and concatenated with the respective DNA polymerase sequences to produce an aa base tree.
Our results showed that the LeHV-5 clearly grouped inside the MuHV-68 like Rhadinovirus, despite presenting a divergence of herpesviruses from rodents, showing a clear phylogenetic separation, which better the quality and relevance of our work. The absence of available sequences from other leporid gammaherpesviruses made it impossible to compare them.
We hope to have now reached the quality standards for publication and hope to see our manuscript published soon in the Plos One Journal.

Kind regards
Fábio Abade dos Santos
