# Supplementary material for: First description of a herpesvirus infection in genus Lepus
Source: PLoS One. 2020 Apr 17;15(4):e0231795. doi: 10.1371/journal.pone.0231795 (PMC7164596; doi:10.1371/journal.pone.0231795)
Supplement: Attachment — Submitted filename: review comments.docx [file pone.0231795.s001.docx]

Review comments for the manuscript number: PONE-D-20-00203

In the present manuscript, authors have identified a new herpesvirus Leporid gammaherpesvirus 5 (LeHV-5), in genus Lepus. They confirmed the presence of herpesvirus in 13 MYXV-positive hares by PCR and sequencing analysis.

However, I would like to suggest authors to pay attention to following points:

1. Table 1 has not been numbered correctly.
2. Table 2 is missing
3. Figure legend must be little more descriptive
4. Figures 6, 7, 8 are missing
5. Bacteriological and parasitological examination method should have had been described in detail under methodology section.
6. Description of figure 2 under the heading Electron microscopy is confusing.
7. Results for epithelial and stroma cell of the eyelid are not shown but discussed. Either show the results or don’t discuss.
