# Supplementary material for: First description of a herpesvirus infection in genus Lepus
Source: PLoS One. 2020 Apr 17;15(4):e0231795. doi: 10.1371/journal.pone.0231795 (PMC7164596; doi:10.1371/journal.pone.0231795)
Supplement: Attachment — Submitted filename: POne_D-20-00203_review.docx [file pone.0231795.s002.docx]

**First description of a herpesvirus infection in genus Lepus**

Abade dos Santos, F.A.1,2*, *Monteiro, M.1, Pinto, A.3, Carvalho, C.L. 1, Peleteiro*

*M.C.*2, Carvalho, P.1, Mendonça, P.1, Carvalho, T.3, Duarte, M.D.1,2

**Author Details**

Abade dos Santos, F.A.1,2 DVM, MSc

Monteiro, M. 1 DVM

Pinto, A. 3,4 BMSc, MSc

Carvalho, C.L.2, DVM, PhD

Peleteiro, M.C.1, DVM, PhD

Carvalho, P.1 DVM

Mendonça, P.1 DVM

Carvalho, T.3, DVM, PhD

Duarte, M.D.2, DVM, MSc, PhD

1Instituto Nacional de Investigação Agrária e Veterinária, Av. da República,

Quinta do Marquês, 2780-157 Oeiras, Portugal

2CIISA, Faculdade de Medicina Veterinária, Universidade de Lisboa, Avenida da

Universidade Técnica, 1300-477 Lisboa, Portugal

3Instituto de Medicina Molecular João Lobo Antunes, Faculdade de Medicina,

Universidade de Lisboa, Avenida Professor Egas Moniz, 1649-028 Lisboa,

Portugal

4 EM Suite, Royal Brompton Hospital and Harefield NHS Foundation Trust.

Manuscript Click here to access/download;Manuscript;Manuscript_PB.pdf

**Corresponding author:**

Abade dos Santos, F.A.

Faculdade de Medicina Veterinária, Avenida da Universidade Técnica 1300-477

Lisbon

Faas@fmv.ulisboa.pt

+351 21 365 2800

**Key words**

Herpesviruses, Gammaherpesvirinae, European hare, *Lepus granatensis*,

Leporids

**Running title :** First Herpesvirus in Iberian hare.

• **Funding**

Most of the field and laboratory work referred to in this manuscript was supported

by the Fundação para a Ciência e Tecnologia (FCT) (Grant

SFRH/BD/137067/2018), Fundo Florestal Permanente (Government of Portugal)

in the scope of the Action Plan for the Control of Rabbit Viral Haemorrhagic

Disease (+COELHO, Dispatch no. 4757/2017 of 31 May) and by the

Interdisciplinary Research Centre on Animal Health, Faculty of Veterinary

Medicine, University of Lisbon (CIISA, FMV-UL) (Portugal) (Project

UID/CVT/00276/2013).

Funding bodies played no direct role in the design or conclusion of the study.

• **Authors’ contributions**

Abade dos Santos, F.A.: Experimental design. Collection of animals,

Necropsies, Sequencing, Histopathology analysis. Wrote the manuscript.

Monteiro, M.: Necropsies, Sequencing, Histopathology analysis and lesion

pattern study.

Pinto, A.: Preformed all the EM analysis.

Carvalho C.L.: Wrote the manuscript.

Peleteiro, M.C.: Histopathology analysis. Revised the manuscript.

Carvalho, P.: Necropsies.

Mendonça, P.: Necropsies.

Carvalho, T.: Interpretation of EM resuts.

Duarte, M.D.: Data collection and analysis. Wrote and revised the manuscript.

• **Acknowledgements**

We thank Sebastião Miguel (Hunting manager), João Carvalho (ANPC), Jacinto

Amaro (FENCAÇA), Fernando Castanheira Pinto (CNCP) and Duarte Nuno

(ICNF) for sample collection and organization of the sampling events. We are

also greateful to Teresa Fagulha (INIAV, Virology Laboratory) for help with

molecular characterization and to Maria João (INIAV, Virology Laboratory) for

technical assistance. Finally we also thank to all the hunters who participated in

field work and sample collection.

**Declarations**

This article didn’t use live animals and was carried out within the scope of a

National Plan for the Control of Rabbit Haemorrhagic Disease Virus 2 in rabbits

(Dispatch no. 4757/2017 of 31 May), with the legal authorizations from the

National Authority, the Instituto da Conservação da Natureza e Florestas (ICNF).

**Abstract**

During the necropsies of Iberian hares obtained in 2018/2019, along with signs of

the nodular myxomatosis form, other unexpected external lesions were also observed.

Histopathology revealed nuclear inclusion bodies in stromal cells suggesting the

additional presence of a nuclear replicating virus.

Electron microscopy further demonstrated the presence of herpesvirus particles in the

tissues of affected hares.

We confirmed the presence of herpesvirus in 13 MYXV-positive hares by PCR and

sequencing analysis. Herpesvirus-DNA was also detected in six healthy hares,

suggesting its asymptomatic circulation.

Phylogenetic analysis based on DNA polymerase gene, grouped this virus within the

gammaherpesvirus cluster, close to herpesviruses from bats and rodents.

We propose to name this new virus Leporid gammaherpesvirus 5 (LeHV-5), according

to the International Committee on Taxonomy of Viruses standards.

The impact of herpesvirus infection on the reproduction and mortality of Iberian hare is

yet unknown but may aggravate the population loss caused by the recently emerged

myxomatosis.

**1. Introduction**

The Iberian hare (*Lepus granatensis*), also known as Granada hare, is an endemic

specie of the Iberian Peninsula whose populations are considered stable by the IUCN

holding a ‘*least concern’* conservation status1.

*Lepus granatensis* is the only hare species found in Portugal and the most widespread

in the Iberia2, therefore, highly relevant for the hunting activity in both countries and

particularly for greyhound racing. Over the years, the Iberian hare has been unaffected

by viral diseases that, alongside environmental and anthropogenic factors, led to the

drastic decline of the wild rabbit. *** Sentence in yellow is missleading

*** *Lepus granatensis* was considered naturally resistant

to myxomatosis, which is endemic in Iberian Peninsula since 19563, despite very sporadic

reports of the disease in the European brown hare (*Lepus europaeus*), namely in France

and Ireland4–6. However, during the summer and autumn of 2018 outbreaks of

myxomatosis in the Iberian hare were reported in Spain7 and Portugal11, respectively.

No other diseases of viral origin have been described in the Iberian hare, including any

caused by herpesviruses that may have a fatal outcome in rabbits.

Until now, four herpesviruses have been identified in leporids: Leporid herpesvirus 1

(LHV-1), Leporid herpesvirus 2 (LHV-2), Leporid herpesvirus 3 (LHV-3) and Leporid

herpesvirus 4 (LHV-4) (Table 1). Of these, the most common naturally occurring

herpesvirus infections identified in rabbits are LHV-2 and LHV-3 (revised on ***???*** Jin et al.,

2008), which alongside LHV-1 belong to the *Gammaherpesvirinae* subfamily.

Conversely, LHV-4 is a member of the *Alphaherpesvirinae* subfamily. These distinct

herpesviruses have a broadly variable impact on the European rabbit, with LHV-2 and

LHV-3 infections usually passing unnoticed, while LHV-4 is far more aggressive, causing

fatal infections.

Herpesviruses are enveloped viruses of 200-250nm in diameter, organised in four concentric layers9, 1) a core with the linear dsDNA genome, 2) T=16 icosahedral capsid

with about 125nm of diameter surrounded by a 3) proteinaceous tegument that contains

many virus-coded proteins and enclosed in a 4) lipid envelope containing several viral

glycoproteins. Morphologically, herpesviruses are distinct from all other viruses10.

Herpesviruses belong to order *Herpesvirales* that comprises three families, namely the

*Herpesviridae* family, which includes more than 100 viruses of mammals, birds and

reptiles and whose members have large genomes ranging from 125 to 290kb11, the

*Alloherpesviridae* family, which includes the fish and frog viruses, and the

*Malacoherpesviridae* family, which contains the bivalve virus10.

The *Herpesviridae* family includes the subfamilies *Alphaherpesvirinae*,

*Betaherpesvirinae* and *Gammaherpesvirinae. Their members*have different biologic properties and distinct classification, supported by phylogeny. The *Gammaherpesvirinae* subfamily is

divided into four genera, namely *Macavirus*, *Percavirus*, *Lymphocryptovirus*, and

*Rhadinovirus*10.

While the subfamily *Alphaherpesvirinae* causes rapid lysis in cell culture, members of

*Betaherpesvirinae* grow slowly inducing the formation of giant cells in culture, and

*Gammaherpesvirinae* typically infect lymphoid tissue, meaning a primary tropism for

lymphoid lineage cells12, which can lead to lymphoproliferative diseases8 and

oncogenesis11. The lack of a suitable animal model ***MHV68 is used as a model virus since many years***

for the *Gammaherpesvirinae*

subfamily, hampers the study of the reactivation mechanisms and pathogenesis of these

oncogenic viruses 13.

In this study, we investigated the presence of herpesvirus in MYXV-positive hares

because during necropsies, along with the typical myxoma virus-induced skin lesions,

other lesions were registered in the genitalia, palpebrae, lips and nose of the animals.

Moreover, in histopathological examination, nuclear inclusion bodies compatible with

herpesvirus were also observed. To investigate the prevalence of herpesvirus in the

hare populations, healthy hares were also tested.

**2. Materials and Methods**

**2.1. Sample**

A total of 38 Iberian hares were investigated within the scope of a national surveillance

program (Dispatch 4757/17, 31th may)14 in action since August 2017. Of these, 16 were

males, 20 females and two failed sex-determination. Eighteen were hunted during the

2018/2019 legal season (October to December), while 20 were found dead in the field

between October 2018 and June 2019. The hares originated from southern Portugal namely

from the districts of Santarém, Beja and Évora.

**2.2. Necropsy and Histopathology**

Cadavers were necropsied and spleen, liver, lung, duodenum and skin samples (namely

scrotum, lips and nose) were collected for virology, bacteriology and histopathology. The

entire gastrointestinal tract was taken for parasitological analysis. From hunted hares,

only spleen, liver and lung samples were received at the laboratory.

For histopathology, skin and genitalia fragments were fixated in 10% neutral buffered

formalin, routinely paraffin embedded, sectioned at 4 μm, and stained with H&E ***spell out***.

**2.3. Transmission electron microscopy**

The fragments selected for transmission electron microscopy (TEM) were formalin

fixated for 48h or on a solution 0.1M sodium cacodylate (Sigma©) containing 2.5%

gluteraldehyde (Sigma©) for 72h. For samples previously embedded in paraffin, the regions

of interest were extracted from the block, sliced smaller than ~1mm3 with a scalpel blade

into two separate viles, and washed thoroughly in xylene. After rehydration using

decreasing concentrations of ethanol, fragments were washed in 0.1M cacodylate

buffer15. Samples were then post-fixed with 2% osmium tetroxide (EMS) for 30min, and

stained in block with 1% Millipore-filtered uranyl acetate (Agar Scientifics), after which

they were dehydrated in increasing concentrations of ethanol, infiltrated and embedded

in EMBed-812 hard (EMS). Polymerization was performed at 60°C for 2 days. Ultrathin

sections were cut either in a UC7 ultramicrotome or in a Reichert ultracut E

ultramicrotome (Leica), collected to 1% formvar coated copper slot grids (Agar scientific),

stained with uranyl acetate and lead citrate (Sigma) and examined in a H-7000

transmission electron microscope (Hitachi) at an accelerating voltage of 100 kV or Jeol

1400plus transmission electron microscope at an accelerating voltage of 120 kV. Digital

images were obtained using a Megaview mid mount digital camera (Olympus) or using

a AMT XR16 bottom mid mount digital camera (AMT©). The sections were systematically

analysed using AMT© software and several high and low magnifications were acquired.

**2.4. Bacteriological and parasitological examination**

Liver, spleen and lung samples were analysed using standard bacteriological methods.

Parasitological examination of the intestine was carried out resourcing to direct wet

mount, sedimentation and filtration techniques.

**2.5. Virological examination**

For nucleic acid extraction, fresh samples of liver and spleen were homogenised at 10%

with phosphate buffered saline (PBS) and clarified at 3000g for 5 min. Total DNA and

RNA were extracted from 200μl of the clarified supernatants, using the MagAttract 96

cador Pathogen Kit in a BioSprint 96 nucleic acid extractor (Qiagen, Hilden, Germany),

according to the manufacturer’s protocol.

All the animals were tested for rabbit haemorrhagic disease virus 2 (RHDV2) and

Myxoma virus (MYXV) by real time PCR systems described before12,22. LHV-4 was

investigated specifically by using the PCR described by Jin et al, 20088. A generalist

nested PCR directed to the herpesviral *DNA polymerase* that allows the detection of

herpesviruses of different subfamilies (Devanter et al. 1996) was also used (Table 2).

Amplifications were carried out in a Bio-Rad CFX96™ Thermal Cycler (Bio-Rad

Laboratories Srl, Redmond, USA), using the One Step RT-PCR kit (Qiagen, Hilden,

Germany) for RHDV2, and the HighFidelity PCR Master Mix (Roche Diagnostics GmbH,

Mannheim, Germany), for MYXV and herpesvirus detection, respectively.

All amplifications were performed as described in Table 2.

**2.6. Sequencing analysis**

The PCR product was visualised in 2% horizontal electrophoresis agarose gel, purified

using the NZYGelpure kit (NZYTECH), and directly sequenced using the ABI Prism

BigDye Terminator v3.1 Cycle sequencing kit on a 3130 Genetic Analyser (Applied

Biosystems, Foster City, CA, U.S.A). Nucleotide forward and reverse sequences were

analysed, assembled into consensus sequences, and submitted to GenBank. The

nucleotide sequences were translated using Mega X 10.1 software.

**2.7. Phylogenetic analysis**

Partial nucleotide and amino acid sequences of the viral *DNA polymerase* were aligned

using the Clustal W with gap opening penalty and a gap extend penalty of 30 and 15,

respectively.

For both sets of data, evolutionary analyses were conducted in MEGA X19.

The model for both phylogenies was selected by *Model function* (MEGA X). LG+G model

according to BIC (4355.57) and AICC (3943.54) criteria was selected for the proteinbased

tree.

The phylogeny partial nucleotide sequences of gammaherpesviruses was inferred by

using the Maximum Likelihood method and Tamura 3-parameter model20. The bootstrap

consensus trees were inferred from 1000 replicates21.

**2.8. Herpesvirus isolation**

Isolation of herpesvirus was attempted from organs of MYXV and herpesvirus coinfected

hares, namely from liver and spleen, penile and vulva samples. In addition, liver

and spleen samples from two hares infected with Herpesvirus alone, were also used.

Samples were homogenized at 20% in phosphate-buffered saline containing penicillin,

streptomycin and amphotericin B (antibiotic-antimycotic), used according to the

manufacturer (Gibco, Life Technologies Corporation). Following centrifugation, the

supernatant was filtered through a 0.45-μm-pore-size filter (Millipore Express) and used

to inoculate sub 70% confluent Candrel R Feline Kidney (CRFK) epithelial cells, Vero

cells and Rabbit Kidney (RK13). RK13 grown in Eagle medium and the others in

Dulbecco's modified Eagle's Media was supplemented with 10% foetal calf serum

(Gibco) and 50μg/ml gentamicin (Gibco). Cells were maintained at 37°C under a

humidified atmosphere with 5% CO2 and observed daily for cytopathic effect (CPE) by

microscopy. Three passages were carried out and the supernatant and cell pellet of each

passage tested for the presence of herpesvirus by PCR.

**3. Results**

**3.1. Necropsy showed lesions compatible with herpesvirus**

**infection in Iberian hares with Myxomatosis**

Overall, MYXV-positive hares revealed poor body condition and, alongside typical

myxomatosis lesions, necrosis of the genitalia (most evident in males affecting the penile

glans but also observed in females, affecting the vulva) was noted in over 70% of the

hares studied. Other lesions observed in these hare specimens, uncommon in rabbits

affected by myxomatosis, included the presence of herpetic-like skin vesicles.

Further investigation of the macroscopic lesions and histopathological patterns was

carried out in hares co-infected with LeHV-5 and MYXV. At this time, we disclose the

macroscopic and histopathological findings from two male hares found dead in

November 2018 (#38455/18) and August 2019 (#25456/19).

Hare #38455/18 presented with eyelids thickened by the accumulation of mucopurulent

exudate and marked enlargement of the penis with irregular surface measuring 1.3x1cm

in diameter (normal diameter is less than 0.5cm) (Figure 1 and Figure 1A) lined with lightyellow

dry exudate.

At the necropsy of hare #25456/19, ulcerated multinodular thickening of the eyelids and

lips was observed. Accumulation of mucopurulent exudate in both eyes was also noted

and a small vesicle was present in the lower lip (Figure 2).

**3.2. Histopathology**

The dermis of hare #38455/18 showed fusiform or stellate mesenchymal cell

proliferation, surrounded by abundant extracellular matrix, scattered infiltration by

lymphocytes and macrophages, and small aggregates of heterophiles, consistent with

myxomatosis.

The penile epithelium of this hare was mostly necrotic and replaced by a thick band of

necrotic cells, heterophils and red blood cells (Figure 1).

Severe heterophile infiltrations of the stroma, in either a diffuse pattern or multifocal

aggregates, were also seen. In the stroma, there was proliferation of pleomorphic spindle

cells, with some nuclei almost completely filled with slightly eosinophilic inclusion bodies

(Cowdry type A inclusions) (Figure 3), suggesting a nuclear replicating virus. These

lesions unexpected from myxomatosis are compatible with herpesvirus.

In the skin of hare #25456/19, a ballooning degeneration of keratinocytes was registered.

Coalescent intra-epidermal and subepidermal vesicopustules (Figure 4) filled with fibrin

and necrotic cells debris and multifocal detachment of the eyelids, lips and foreskin

epidermis were seen. In the underlying dermis, multifocal haemorrhages, intense

infiltration by heterophils and necrotic cells with accumulation of chromatin debris were

present (Figure 4).

Below the dermis, accumulation of myxoid tissue with pleomorphic spindle cells, some

of which showing rounded or oval and slightly eosinophilic intranuclear inclusion bodies,

was observed (Figure 5). An infiltrate of mononucleated inflammatory cells and

heterophils was present in skeletal muscle tissue.

**3.3. Electron microscopy**

Samples from hares #38455/18 and #25456/19, were further processed and analysed

for TEM allowing the confirmation of the presence of herpesvirus in different tissues.

In the penile soft tissue of hare #38455/18 (Figure 6), spherical virions, with structure

and dimensions compatible with herpesviruses, comprising an inner core packed into an

icosahedral capsid, were observed in the nucleus of stromal cells, indicating nuclear

replication which is an attribute of herpesviruses (Figure 2). The viral capsid contained a

relatively small, asymmetrical, electron-dense region, which probably represents the

condensed DNA core. In this animal, also positive to myxomatosis, no MYXV particles

were found in the samples processed.

In the case of hare #25456/19, herpesviruses were visualised in epithelial and stroma

cells of the eyelid (*results not shown*).

**3.4. Virological, bacteriological and parasitological results**

None of the 38 hares investigated in this study tested positive to RHDV, RHDV2 or LHV-

4. Fifty percent of the hares were positive to LeHV-5, of which 68.4% (13/19) were also

positive to MYXV.

Herpesvirus-DNA was also detected by PCR in the liver, spleen and lung samples of

apparently healthy hares that tested negative for MYXV (n=7). From these hunted

specimens***what is this?***, no genitalia/skin samples were available for histopathology. Six hares

showed doubtful results.

Parasitological and bacteriological examinations did not reveal any infections that could

justify the death of these animals.

**3.5. Molecular characterisation of Iberian hare herpesvirus**

For 19 of the 38 animals, an amplicon 225 bp-long, compatible with herpesvirus, was

obtained in the pan-herpesvirus PCR, while for six samples only a weak band was

generated. The presence of herpesvirus was confirmed by sequencing analysis in 16

hares. For 11 of these amplicons, the reads obtained were independently assembled

and edited, showing 100% similarity to each other. Blast analysis of the sequences

confirmed that the obtained fragment is part of the herpesvirus DNA polymerase coding sequence.

Comparison with other herpesvirus sequences available in the GenBank database

(03.07.2019), showed similarity, with 46% of query cover and 81.73% of identity, with an

unidentified herpesvirus (KT591396.1) obtained from *Rattus norvegicus*.

Five of the obtained sequences were submitted to the GenBank (

accession numbers MN557129, MN557130, MN557131, MN557132 and MN557133).

**3.6. Phylogenetic analysis**

The region amplified by the nested PCR has discriminatory power to allow phylogenetic

inferences18. To investigate the phylogenetic relationship of the Iberian hare

herpesvirus found in this study (represented by sequence MN557129), with other

members of the *Herpesviridae* family, a set of 37 DNA polymerase protein sequences

from alpha-, beta- and gammaherpesviruses, obtained from Genbank, were edited to

span a 68 aa-residue region.

Unrooted Maximum Likelihood tree showed that the herpesvirus sequence from *Lepus*

*granatensis* clearly groups within the gammaherpesvirus cluster (Figure 7). MN557129

appears to share a common ancestor with gammaherpesviruses from *Scotophilus kuhlii*

(Genbank access numbers ALH21079.1, KR261869.1 and ALH21071.1), a small

insectivore bat native of Asia and India, as well as with some rodent herpesviruses (Genbank accession

numbers KT591396.1, KT591394.1 and others) despite supported by a low bootstrap

value (<25) (Figure 7).

To refine the phylogenetic analysis with regards to gammahespesviruses (Figure 8), we

explored the nucleotide variability among this group, using a second set of 25

herpesviruses sequences from orders Artiodactyla, Carnivora, Chiroptera, Lagomorpha,

Perissodactyla, Primates and Rodentia. The accession numbers of the original sequences

from which the *DNA Polymerase* 225 nt ***The PCR products are of appr. 225 bp length. But after subtraction of the primer sequences, the novel sequence has a length of appr 175 bp only !*** long sequences were edited, are indicated in

Figure 8.

This phylogenetic analysis confirmed that the leporid herpesvirus under study is a

gammaherpesvirus, more closely related to herpesviruses from *Scotophilus kuilii*, of

unclassified genera.

According with the most recent International Committee on Taxonomy of Viruses (ICTV)

guidelines for classification of viruses, we propose to name this virus species leporid

gammaherpesvirus 5 (LHV-5), following the rabbit alphaherpesvirus 4 (LHV-4),

although we cannot suggest a genus for LHV-5. ***None oft he LHVs is currrently classified as species by ICTV***

**3.7. Isolation of the viruses in cell cultures**

The fact that LeHV-5 is a gammaherpesvirus may explain the difficulties found in viral

isolation, which was not achieved with either of the two strategies adopted.

Despite LeHV-5 not appearing to multiply in human (Hela) and primate (Vero) cells,

because gammaherpesviruses may be zoonotic, as is the case of cercopithecine

herpesvirus-1 and murine gamma herpesvirus13***??? Cercopithecine herpesvirus-1 is in the Alphaherpesvirinae! There is no zoonotic murine gammaherpesvirus

***, isolation attempts was performed under

BSL-2 conditions.

**4. Discussion and Conclusion**

This study describes the detection of a new gammaherpesvirus in the genus Lepus *** Please rephrase

*** that, according tophylogenetic analysis, is most similar to bat and rodent gammaherpesviruses.

Viral isolation was not accomplished in RK13, CRFK or Vero cells. The difficulty in

growing the virus *in vitro*, an important step towards its characterisation, may be related

to the co-infection with MYXV. The greater ease in multiplication of MYXV, which grows

in many cell cultures22,23, may hamper herpesvirus isolation. Moreover, wild animal

samples are frequently somewhat autolysed and usually frozen before reaching the

laboratory, which may lead to herpesvirus inactivation. The complexity in isolating genital

gammaherpesvirus in cell culture was also referred by Saliki et al. (2006). The lack of

cell cultures from Lepus species may pose further challenges.

Our investigation revealed the presence of necrosis of the genitals and herpetic-like

vesicles in the lips of hares co-infected with LeHV-5 and MYXV, which were attributed to

herpesvirus.

Notwithstanding the sampling limitation, the fact that macroscopic lesions were only seen

in animals with myxomatosis, suggests that MYXV may play a role on herpesvirus

replication and/or reactivation by compromising the immune response of the host,

leading to the development of clinical disease with exuberant lesions.

Immunosuppression facilitates herpesvirus infections and virus reactivation, and it was

demonstrated that certain MYXV proteins, such as Serp-1, have strong immune

suppressing effects23. MYXV infection may hence represent a stress and/or

immunosuppressive triggering factor for herpesvirus infection. It is known that stress, disease and

other factors such as extreme temperatures can lead to the resurgence of herpesviruses

in other species25. It was observed that depressed T-cell immune function reduces the

quality of immune surveillance resulting in the increase of viral activity 13.

Herpesviruses generally follow one of three distinct strategies13 within the host, namely

i) latency with occasional re-emergence, ii) hit-and-run’ approach and iii) slow-and-low’

tactic 13.In the case of LeHV-5, because it affects wildlife, specimens are mainly animals

found dead or moribund, limiting the conclusions on the strategy of the virus.

In addition, herpesviruses are frequently found either in the absence of clinical signs or

in association with very diverse clinical signs26.This fact complicates the understanding

of the true role and contribution of many herpesviruses in the courses of certain diseases,

especially with regards to wild species that are often exposed to, and infected by, many

pathogens. On the other hand, animal experimentation is complicated by the absence of

available specific pathogen free (SPF) specimens, and by the difficulties in keeping hares

in captivity, limiting cause-effect experiments. Moreover, during latency, herpesviruses

may not be detected by current methods, as it is the case of gammaherpesvirus in horses, resulting in

an underestimated prevalence in the populations26,27.Reports indicate that equine

herpesvirus 2 (EHV-2) can be detected in immuncompetent animals in the absence of

signs of disease (revised on Marenzoni et al., 2015), meaning that healthy animals can

be a potentially source of viral transmission.

According to our study, based on viral DNA amplification, around half of animals tested

(63% symptomatic and 37% asymptomatic) were positive for LHV-5. However, this

value may be an underestimation given that the tropism of LeHV-5 is still unknown, and

consequently the tissue samples used for diagnosis may have been inadequate ***for what?***. Thus we cannot assure that PCR-negative animals were not infected by LeHV-5.

The fact that herpetic lesions were not observed in young, could mean that if the

acquisition of LeHV-5 occurs at an early age, primary infection takes place with mild or

no symptoms. In the absence of an unbalancing triggering factor such as MYXV

infection, LeHV-5 may successfully establish a long-term relationship with the hare host,

with subclinical disease and transient viremia. This would explain the detection of

herpesvirus DNA in apparently healthy hares.

Interestingly, the gammaherpesvirus identified in external genitalia of the investigated

hares was not associated with the development of papillary lesions as in other genital

gammaherpesvirus infections24. However, given that hunters, hunting managers and

landowners have the opinion that the reproduction of the Iberian hare has been declining

in recent years, it is important to clarify if this reduction is also associated with the

emergence or circulation of LeHV-5. Other important concern is the potential production

of oncogenic proteins by this herpesvirus.

According to our findings, genital herpesvirus may have a critical effect on hares’ fertility

and reproduction as well as in their survival. Hence, it is crucial to evaluate and

understand the extent to which MYXV plays a role in the infection/reactivation of

herpesvirus, as well as the putative role of herpesvirus in favouring infection of hares by

MYXV or aggravating the severity of myxomatosis clinical forms.

The virological results obtained in this study also disclosed the infection of apparently

healthy hares by LeHV-5, suggesting the possible circulation of this virus in the wild

populations in a systemic subclinical form.

Although Iberian hare populations are still considered stable, no census is available for

the hare populations in Portugal.

Presently, we continue monitoring apparently healthy and MYXV-positive hares in

mainland Portugal to determine the extent of the geographic distribution of LeHV-5

among the wild hare populations, and the putative association of herpesviruses with the

virulence of the recently emerged hare myxoma virus.

It is important to evaluate the geographical distribution of the virus, the epidemiology of

the disease, the real extent and severity of the lesions induced in hares by LeHV-5, the

persistence and latency of herpesvirus in the wild populations and the LeHV-5-MYXV

associated pathology in order to predict the consequences of the LeHV-5 infection at

population level and to evaluate its importance in the future of this iconic species.
